# The Surgical Method of Craniectomy Differentially Affects Acute Seizures, Brain Deformation, and Behavior in a Traumatic Brain Injury Animal Model

**DOI:** 10.1089/neur.2024.0064

**Published:** 2024-10-07

**Authors:** Cesar Santana-Gomez, Gregory Smith, Ava Mousavi, Mohamad Shamas, Neil G. Harris, Richard Staba

**Affiliations:** ^1^ Department of Neurology, David Geffen School of Medicine at UCLA, Los Angeles, California, USA.; ^2^ Department of Neurosurgery, David Geffen School of Medicine at UCLA, Los Angeles, California, USA.

**Keywords:** acute seizure activity, craniectomy method, EEG, traumatic brain injury

## Abstract

Traumatic brain injury (TBI) is the leading cause of morbidity and mortality worldwide. Multiple injury models have been developed to study this neurological disorder. One such model is the lateral fluid percussion injury (LFPI) rodent model. The LFPI model can be generated with different surgical procedures that could affect the injury and be reflected in neurobehavioral dysfunction and acute electroencephalograph (EEG) changes. A craniectomy was performed either with a trephine hand drill or with a trephine electric drill that was centered over the left hemisphere of adult, male Sprague Dawley rats. Sham craniectomy groups were assessed by hand-drilled (Sham_HMRI_) and electric-drilled (Sham_EMRI_) to evaluate by magnetic resonance imaging (MRI). Then, TBI was induced in separate groups, (TBI_H_) and (TBI_E_), using a fluid-percussion device. Sham-injured rats (Sham_H_/Sham_E_) underwent the same surgical procedures as the TBI rats. During the same surgery session, rats were implanted with screw and microwire electrodes positioned in the neocortex and hippocampus and the EEG activity was recorded 24 h for the first 7 days after TBI for assessing the acute EEG seizure and gamma event coupling. The electric drilling craniectomy induced greater tissue damage and sensorimotor deficits compared with the hand drill. Analysis of the EEG revealed acute seizures in at least one animal from each group after the procedure. Both TBI and Sham rats from the electric drill groups had a significant greater total number of seizures than the animals that were craniectomized manually (*p* < 0.05). Similarly, EEG functional connectivity was lower in Sham_E_ compared with Sham_H_ rats. These results suggest that electrical versus hand-drilling craniectomies produce cortical injury in addition to the LFPI which increases the likelihood for acute post-traumatic seizures. Differences in the surgical approach could be one reason for the variability in the injury that makes it difficult to replicate results between preclinical TBI studies.

## Introduction

The lateral fluid percussion injury (LFPI) is a well-studied model of human traumatic brain injury (TBI),^1–3^ and has been used in rabbits, cats, rats, mice, and pigs.^4–8^ The injury is produced by applying a short duration fluid pulse (∼20 msec) through a craniectomy against an intact, exposed dura, resulting in a transient deformation of the brain. The LFPI model can produce graded neurological, histological, and cognitive outcomes that replicate those seen in human TBI,^9,10^ and in cases of moderate-to-severe LFPI, acute, and sometimes late post-traumatic seizures.^11,12^

As with all experimental models, modifications are made to the LFPI rat model depending on the surgeon’s technique and experience, and/or goals of the research. For example, some studies report performing the craniectomy with a dental electric drill adapted with jeweler’s bur or trephine,^12,13^ whereas others report using a hand-drill trephine.^14^ Studies show an extensive network of blood vessels and nerves between the rat brain and calvaria^15^ and craniectomy alone, especially when performed with an electrical drill, can disrupt this network, resulting in an upregulation of proinflammatory proteins,^16–18^ leaky blood vessels,^18–20^ and cerebral edema.^17^ Under these conditions, it is reasonable to predict LFPI with an electric-drilled craniectomy, which can generate measurable heat, vibration, and axial force to the underlying tissue,^18^ and it would be more severe than one with a hand-drilled craniectomy. However, the potential differences in severity during the acute period after LFPI with respect to magnetic resonance imaging (MRI) morphology, electroencephalograph (EEG) functional changes, and behavior are not well-documented.

To address this knowledge gap in the LFPI model, in the current study we performed a series of experiments to first evaluate the MRI morphological and EEG functional connectivity changes associated with an electric-drilled versus hand-drilled craniectomy. Then, in separate cohorts of rats, we performed the same craniectomy procedures in LFPI and sham-injured rats and assessed each for sensorimotor performance up to 28 days after injury and acute post-traumatic seizures during the first 7 days after injury.

## Material and methods

### Experimental overview

**FIG. 1.**
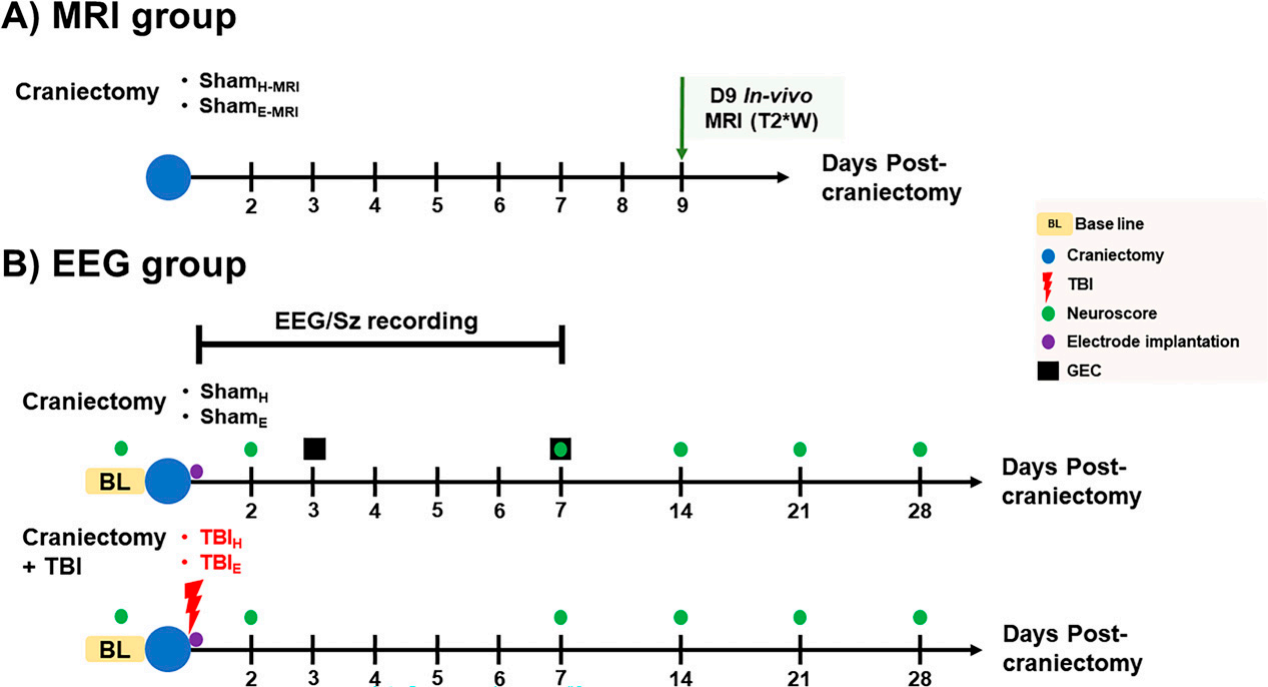
Experimental groups and timeline. The rats were randomized into MRI and EEG groups. **(A)** The MRI group assessed the brain structural changes induced by two different craniectomy methods (denoted by blue circle on timeline) on sham-operated animals (Sham_H-MRI_, Sham_E-MRI_). The animals were imaged on day 9 postcraniectomy. **(B)** In the EEG group, the effect of craniectomy on EEG function connectivity was assessed (top part; Sham_H_, Sham_E_). Electrodes (purple circle) were implanted after craniectomy during same surgery. Functional connectivity was computed as GEC (black square) on days 3 and 7 postcraniectomy. In a separate set of experiments, the effects of craniectomy and TBI on sensorimotor deficits (Neuroscore; green circles) and early seizures (bottom part; TBI_H_, TBI_E_). Neuroscore tests were performed at six time points, including baseline (BL) and EEG was reviewed for early seizures during first 7 days postcraniectomy. EEG, electroencephalograph; GEC, gamma event connectivity; MRI, magnetic resonance imaging; TBI, traumatic brain injury; Sz, seizure.

Adult male Sprague Dawley rats (*n* = 50, 300–350 g at the time of the craniectomy; Charles River Laboratories Inc.) were randomized into two groups for MRI only and four groups for EEG studies (Fig. 1). The rats were housed in individual cages in a controlled environment (temperature, 21–26°C, humidity 30–70%, lights on 06:00–18:00 h) and had free access to food and water. All animal procedures were approved by the University of California Los Angeles Institutional Animal Care and Use Committee (protocol 2000–153-61A).

### Surgery

**FIG. 2.**
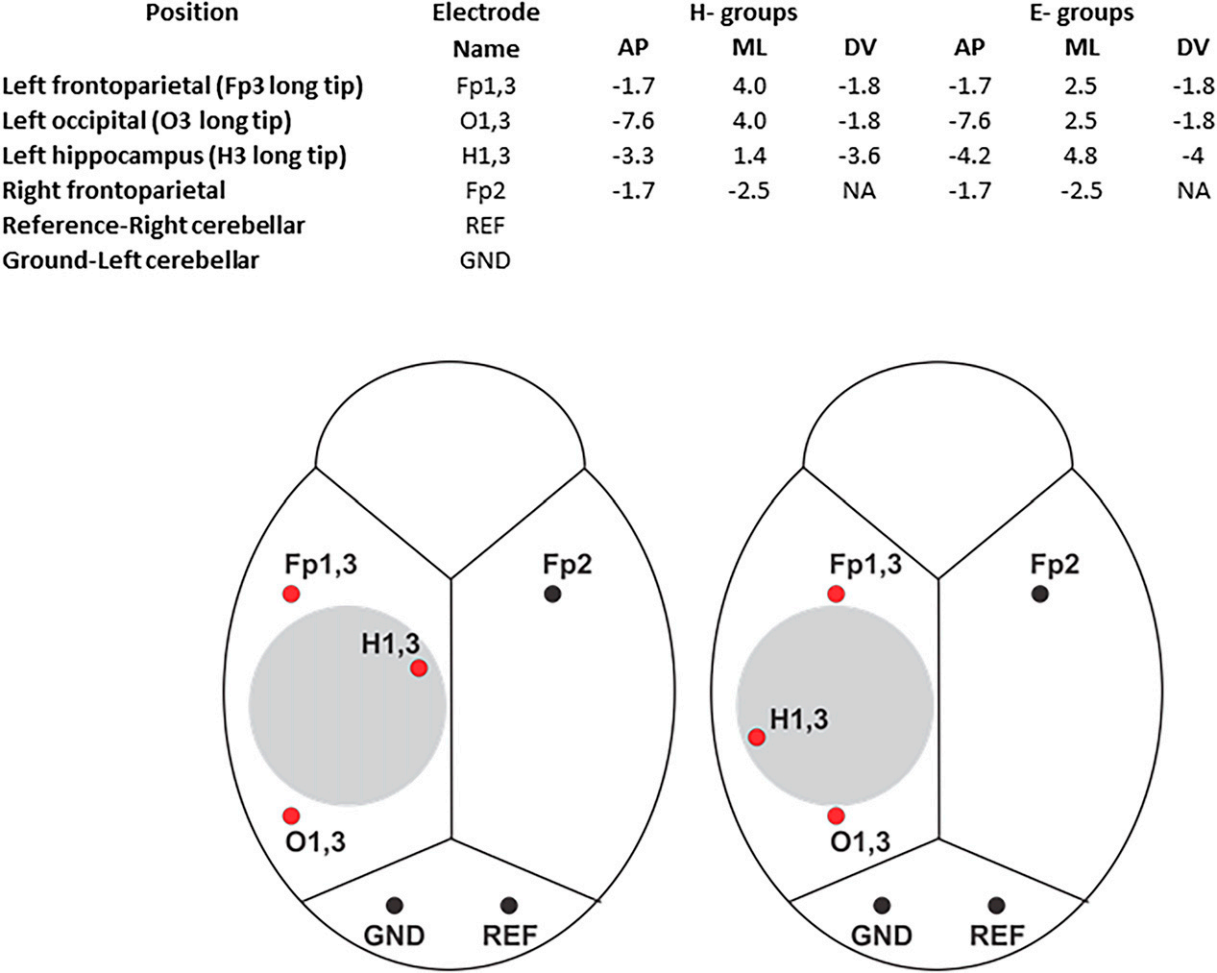
Electrode placements for EEG recording (adapted from Paxinos & Watson).^21^ Coordinates associated with each craniectomy method, which were slightly modified according to the experimental protocol (upper panel). Electrodes were positioned anterior and posterior to the Ø 5 mm craniectomy (grey circle, lower panel), within hippocampus, and over the contralateral hemisphere. Black dots indicate screw electrodes and red dots indicate bipolar penetrating electrodes. Fp1,3, bipolar ipsilateral frontoparietal; Fp2, contralateral frontoparietal; GND, Ground; O1,3, bipolar ipsilateral occipital; H1,3, bipolar hippocampus; REF; Reference; AP, Anterior-Posterior; ML, Medial-Lateral; Dorsal-Ventral.

All rats were anesthetized using 5% isoflurane. The fur atop the rat’s head was shaved, the skin scrubbed with antiseptic, and the rat was draped for surgery. A 3–4 cm midline incision was made, with the skin parted, and the exposed underlying skull was cleaned and dried. In all cases, the craniectomy was positioned over the left parietal bone and centered AP −4.5 mm from bregma and ML +2.5 mm from the sagittal suture (Fig. 2).^21^ The craniectomy was performed using a stainless-steel trephine drill bit (Ø 5 mm; Meisinger, Germany) that was either mounted to a fine pin vise hand drill (Tamiya, Inc., Japan) or single-speed Dremel MultiPro electric drill (35,000 rpm; Dremel US, Mt. Prospect, IL). Trephination used light, but steady, pressure, and alternated between drilling and irrigating with room temperature sterile saline to remove bone dust and reduce bone heating, and was completed in about 30 sec. After the bone was removed, the dura was inspected to ensure it was intact, and the incision was closed using nonabsorbable surgical suture (4–0 nylon suture, Ethicon). The surgical procedures for the MRI and EEG cohorts were performed by two surgeons (C.S.G. and G.S.) with comparable experience who had gone through the same surgical trainings, ensuring consistency in the procedures performed.

#### MRI acquisition, postprocessing, and analysis

**FIG. 3.**
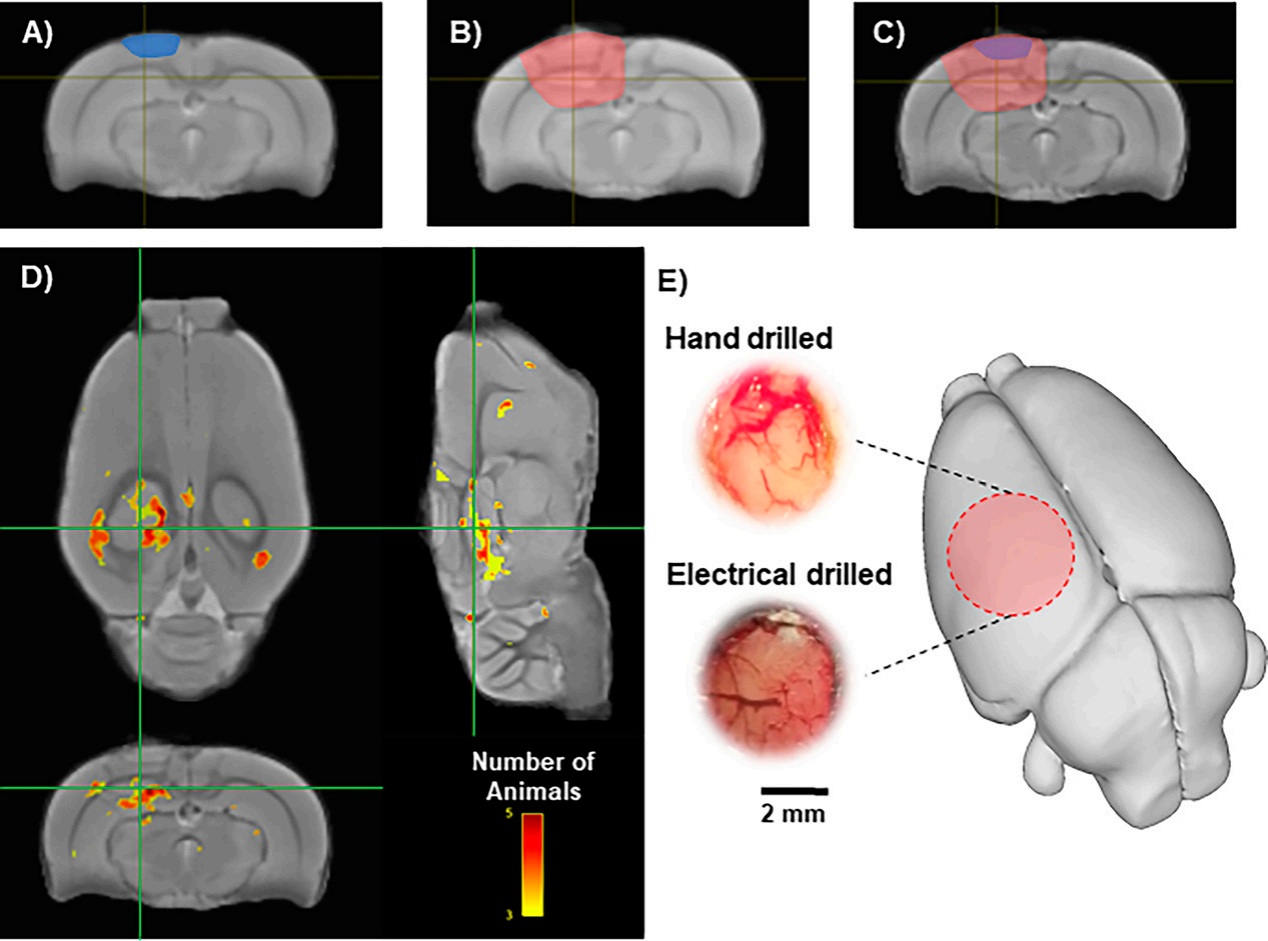
T2* weighted MRI comparison in sham-injured rats. **(A–B)** The depth of tissue damage highlighted over the mean T2* weighted images of the hand-(A; blue-shaded area) and (B; red-shaded area) electrical-drilled craniectomy. **(C)** Overlap of tissue damage between the hand- and electrical-drilled craniectomy superimposed on the mean template of all brains. **(D)** Images illustrate brain regions associated with positive tissue local tissue deformation that was greater in electrical-drilled than hand-drilled craniectomies. Color intensity represents the number of brains altered in that region. Background image is a T2* weighted mean of all brains used in this comparison (Sham_HMRI_, *n* = 5; Sham_EMRI_, *n* = 5). **(E)** Macroscopic view after craniectomy method by hand (top) or electrical drill (bottom) craniectomy method in sham-injury animals. Photographs show darker dura and blood vessels within the craniectomy produced by electrical drill compared with the hand drill. Red circle represents the position of craniectomy site.

To assess the effects of the craniectomy on brain tissue, MRI studies were performed 9 days after surgery in five rats that had craniectomy with hand drill (Sham_H-MRI_) and another five rats with craniectomy using the electrical drill (Sham_E-MRI_). MRI images were acquired using a Bruker Biospin, 7T scanner and a 400 mT/m gradient with a 110 µs rise-time and an actively decoupled, quadrature, surface coil. For anatomical scans a multiecho, gradient echo, three-dimensional sequence was used with a TR of 125 msec, 13 echos, and an effective TE of 2.8–52 msec with a 4.08 msec spacing, a 20° flip angle, and a data matrix of 160 × 122 × 80 mm. Resulting image resolution was 160 mm^3^ isometric. The 13 echoes were averaged to create a T2* weighted map for each rat which was normalized for signal intensity and used to generate a population mean deformation template from rats in all groups using advanced normalization tools (ANTs).^22^ The warp transformation parameters generated by this procedure were then used to obtain a Jacobian determinant map for each rat that reflects local tissue deformation because of the sham or injury procedures. Regions of greatest increase or decrease in tissue formation were found in each brain using population-based, voxel-based statistics by defining a boundary Z score threshold at Z > 2.33 (*p* < 0.01) calculated using the average Sham_H-MRI_ Jacobian determinant maps and its standard deviation. The Sham_H-MRI_ group was chosen because our hypothesis and data suggested this group would have the least tissue deformation. The voxels surviving this threshold were binarized and summed across all rats/group to create the population count overlay map (Fig. 3D). The volume of thresholded voxels was taken from each of the Sham_E-MRI_ rats as a measure of significant positive tissue deformation when compared with the amount of tissue deformation in the Sham_H-MRI_ rats.

### Induction of LFPI

To evaluate the effects of craniectomy in the LFPI model of human TBI, craniectomies were performed (see surgery section) in 13 rats that underwent craniectomy using the hand drill (TBI_H_) and another 14 rats that underwent craniectomy using the electrical drill (TBI_E_). For the LFPI induction, a modified Luer-Lock syringe cap was anchored over the craniectomy and set with dental acrylate. A severe TBI was induced with a fluid percussion injury pendulum device equipped with a straight-tip attachment (AmScien Instruments, Model FP 302, Richmond, VA, USA).^23^ Sham-injured control rats for hand drill (Sham_H_, *n* = 6) and electrical drill (Sham_E_, *n* = 7) craniectomies underwent the same surgical procedures as the TBI rats, but LFPI was not performed.

### Microelectrode implantation and EEG recording

EEG recordings were performed in the TBI and Sham groups using microelectrodes and epidural screw electrodes implanted during the same surgery for LFPI. Paired microwires (Ø 40 µm) with tip separation of 0.5 mm were implanted in the perilesional fronto-parietal cortex (Fp1,3), posterior to the craniectomy in occipital cortex (O1,3), and ipsilateral anterior hippocampus (H1,3). One screw electrode was implanted into contralateral fronto-parietal cortex (Fp2). Ground (GND) and reference (REF) electrodes (stainless steel screws) were inserted in the skull bone overlying the cerebellum. Electrode locations are summarized in Figure 2. The electrodes were mounted in a 12-channel Plastics One pedestal (M12P, PlasticsOne Inc.). The pedestal was secured to the skull with dental acrylic. Sham-injured controls underwent the same electrode implantation procedures as TBI rats.

### EEG data acquisition

Immediately following surgery, the rats were placed in a Plexiglas cage with temperature-regulated (∼37°C) and watched during recovery before being returned to the home Plexiglas cylinder (about 30 min). The electrode headset atop the rat skull was connected to a 12-pin swivel commutator (SL12C; PlasticsOne Inc.) via a flexible shielded cable (M12C-363/2; PlasticsOne Inc.), allowing the rat to move freely during recording. The commutator was connected to an amplifier using a flexible shielded cable 363/2-441/12 (PlasticsOne Inc.). Electrical brain activity was recorded using an Intan RHD200 amplifier and began within an hour after completion of the surgery and continued 24 h/day for the first week after TBI. EEG was recorded with respect to REF electrode overlying the cerebellum and sampled at a minimum of 2 kHz per channel and band-passed between 0.1 Hz and 1 kHz.

### Postinjury functional impairment evaluation

Postinjury sensorimotor deficits were assessed based on the composite neuroscore as previously described.^24^ Rat responses were nominally scored from 0 (severely impaired) to 4 (normal) for (i) left and right contraflexion, (ii) left and right hindlimb flexion, (iii) left and right lateral pulsion, and (iv) ability to stand on an inclined board in a vertical and horizontal (left and right) position. The maximum possible score was 28. Baseline neuroscore was performed 2 days before injury and then 2 days after injury and then each week for the first month. For the baseline inclined board assessment, the angle at which the rat was able to keep a steady posture was given a maximal score of 4. For the postinjury incline board assessment, the injury score was computed as the difference in angle between postinjury and baseline periods (4 = no difference in the angle; 3 = 2.5° less than the baseline; 2 = 5° less than the baseline; 1 = 7.5° less than the baseline; 0 = 10° less than the baseline).^24^

### Seizure detection

The EEG recordings were exported into European data format (EDF) and then analyzed using EDF browser software (https://www.teuniz.net/edfbrowser/). EEG was reviewed using a 90 Hz low pass filtered displayed in 2 min windows. An acute (≤7 days postinjury) electrographic seizure was defined as an event consisting of repetitive epileptiform EEG spike discharges >2 Hz with quasi-rhythmic, spatial, and temporal changes in frequency, amplitude, morphology lasting at least 10 sec.^25^

### Data analysis for gamma event coupling

Gamma event coupling (GEC) in Sham_H_ and Sham_E_ was computed from a 10-min episode of EEG with high-amplitude, irregular activity typical of slow wave sleep on days 3 and 7 after surgery. Episodes were selected that were free of movement or obvious muscle-related artifacts or channels with power line noise. Briefly, the EEG was down sampled to 1 kHz and bandpass filtered between 30 and 55 Hz. Then, the maximum peak of each gamma wave was detected and the lead or lag between gamma maxima from each unique pair of electrodes was quantified using peri-event histograms. Shannon entropy was used to evaluate each histogram and a connectivity factor was calculated by subtracting the observed entropy from the computed maximum entropy and dividing the difference by the computed maximum entropy. These calculations produced a connectivity factor between 0 and 1, where 0 means fully disconnected and 1 means fully connected. Connectivity factors were arranged in symmetrical connectivity matrices where the intersection between a row and column represent the connectivity between a pair of electrodes. To compensate for data loss of noisy channels, a recovery algorithm was used to interpolate the connectivity factor if (1) only one electrode was missing in a rat and day, (2) this electrode was missing in day 3 or 7 but not both days, and (3) data from the electrode was available for at least 2 rats in the same group on both days. To recover the missing data, the percentage of change in connectivity strength between day 3 and 7 was calculated for the missing electrode in all rats in the same group. Because the data were available for 1 day in the considered rat, the average percentage of change was applied to interpolate the pairwise connectivity on the day when the electrode data was missing.

### Statistical analysis

Data analysis was performed using GraphPad Prism (V. 7.03). First, all data were assessed for normal distribution using D’Agostino–Pearson’s omnibus normality test. If not normally distributed, then a nonparametric test was used to compare the variable. The chi-square test was used to assess differences in postimpact parameters and percentage of rats with acute EEG seizures. The differences in angle, pressure, apnea, and righting reflex were calculate using *t*-test. Neuroscore and seizure-related differences were analyzed using the one-way analysis of variance (ANOVA) test followed by *post hoc* analysis with Bonferroni correction. The GEC connectivity value of each pair of channels represented in the upper half of the connectivity matrix for rats in the Sham_E_ group was compared with the corresponding pair of channels in the Sham_H_ group using nonparametric Wilcoxon two-tailed test as the data were not normally distributed. The elements in the connectivity matrices were not treated as independent characteristics as they are coming from the same brain for each sample and hence the statistical comparison was corrected for multiple comparisons using false discovery rate (FDR) correction scheme to eliminate the possibility of statistical difference due to chance and to ensure that it’s directly related to the experimental protocol. The difference was considered significant at *p* ≤ 0.05.

## Results

### Craniectomy

Visual inspection of the brain’s surface through the craniectomy verified that the dura was intact in all rats (Fig. 3). In rats that had a craniectomy performed using a hand drill (Sham_H_ and TBI_H_ groups), the underlying brain tissue appeared light pink in color and the blood vessels presented a red color (Fig. 3E). In contrast, in rats that had a craniectomy using the electric drill (Sham_E_ and TBI_E_ groups), the underlying brain tissue had a dark red, brown hue, and the blood vessels were dark red (Fig. 3E). There was no evidence of bleeding from large vessels on the brain surface in hand- or electric-drilled rats.

### MRI abnormalities after craniectomy

MRI was performed to evaluate the effects of hand- and electric-drilled craniectomies on the underlying brain. The amount of positive tissue deformation was greater in the electrical-drilled craniectomy animals (Sham_E-MRI_) compared with the Sham_H-MRI_ group (Figs. 3A–C). When compared to the Sham_H-MRI_ group, the average volume of tissue that was significantly deformed in the Sham_E-MRI_ group was 34.7 ± 21.6 mm^3^. Deformed tissue was identified on a voxel-by-voxel basis and was considered deformed if the voxel had a Z > 2.33 when compared with the Sham_H-MRI_ group. The location of the significant voxels showing deformation was in an area directly under the craniectomy in most of the Sham_E-MRI_ rats (Fig. 3D).

### Reduced EEG functional connectivity after electric-drilled craniectomy

**FIG. 4.**
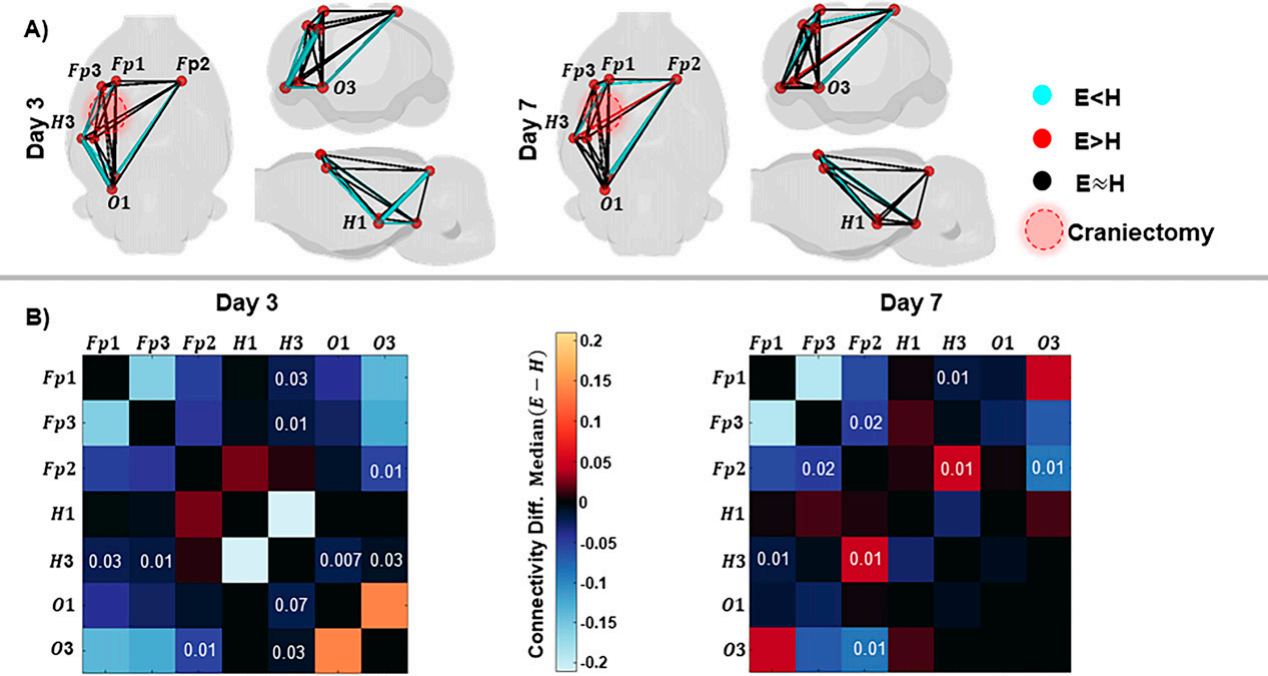
Differences in functional connectivity between Sham_E_ and vs. Sham_H_ rats. **(A)** Connectivity computed as GEC (30–55 Hz) between recording electrodes or nodes (red spheres) on days 3 (left) and 7 (right) after surgery. Differences in the median connectivity between Sham_H_ and vs. Sham_E_ rats represented by the color and proportional to the thickness of lines between nodes. The cyan color denotes weaker connectivity in Sham_E_ than Sham_H_ group, whereas red denotes stronger GEC in Sham_E_ than Sham_H_ group, and black lines indicate no difference. Results depicted in axial, coronal and sagittal views. The injury site is represented as a translucent red circle on the axial view. **(B)** Adjacency matrices of the differences in median connectivity between Sham_E_ and Sham_H_ groups on day 3 (left) and day 7 (right) after surgery. Value at the intersection of the row and column represent the *p* value (<0.05) from the Wilcoxon test for corresponding electrode pairs. Note differences in connectivity were not statistically significant after FDR correction.

In a separate cohort of sham-injured rats, GEC (30–55 Hz) was computed to assess the effects of the craniectomy procedure on EEG functional connectivity. Three days after surgery, Sham_E_ rats had weaker GEC between ipsilateral hippocampus and prefrontal cortex (H3-Fp1, *p* = 0.03; H3-Fp3, *p* = 0.01), as well as to occipital cortex (H3-O1, *p* = 0.007; H3-O3, *p* = 0.03) than Sham_H_ rats (Fig. 4, uncorrected for multiple comparisons). Also, Sham_E_ rats had weaker GEC between contralateral prefrontal cortex and ipsilateral occipital cortex than Sham_H_ rats (Fp2-O3, *p* = 0.001). Seven days after injury, Sham_E_ rats still had weaker GEC between ipsilateral hippocampus and prefrontal cortex (H3-Fp1, *p* = 0.01), and between contralateral prefrontal cortex and ipsilateral occipital cortex (Fp2-O3, *p* = 0.01) and prefrontal cortex (Fp2-Fp3 *p* = 0.02) than the Sham_H_ rats. At this same time, Sham_E_ rats had stronger GEC between ipsilateral hippocampus and contralateral prefrontal cortex than the Sham_H_ rats (H3-Fp2, *p* = 0.01; Fig. 4).

### Induction of LFPI

The mean pendulum angle used for the LFPI to produce severe TBI was 19.8 ± 0.8° in TBI_H_ rats and 17.8 ± 1.3° in TBI_E_ rats (*p* < 0.05; Table 1). The larger angle did not affect the impact pressure between the two groups (*p* > 0.05) but was associated with a longer mean postimpact apnea (*p* < 0.05). After recovery from impact, electrodes were implanted at the coordinates listed in Figure 2. In sham-injured or TBI groups, there was no difference in mortality between hand- versus electric-drilled craniectomy. TBI_H_ and TBI_E_ groups showed higher proportion of rats that died after injury compared with Sham_H_ animals (54% and 57% respectively, chi-square test *p* < 0.05; Table 1).

**Table 1.** Traumatic Brain Injury Rat Model Details

Group	Angle (°)	Pressure (atm)	Apnea (s)	Mortality rate (%)
Sham_H_	—	—	—	0/6 (0%)
Sham_E_	—	—	—	1/7 (14.3%)
TBI_H_	19.8 ± 0.8	2.4 ± 0.1	31.3 ± 18.4	7/13 (53.9%)^*^
TBI_E_	17.8 ± 1.3^**^	2.3 ± 0.1	24.6 ± 10.4^**^	8/14 (57.2%)^*^

^*^ *p* ≤ 0.05 vs. Sham_H_, chi-square test.

^**^ *p* ≤ 0.05 vs. TBI_H_, *t*-test.

### Neuroscore test

**FIG. 5.**
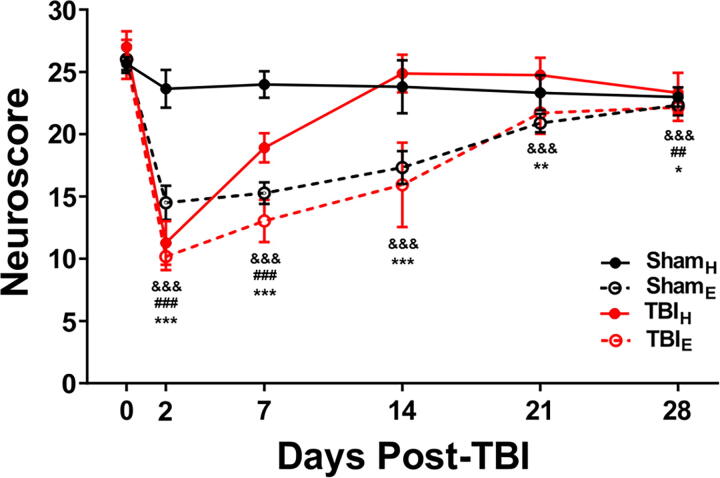
Neuroscore values shows the progression of sensorimotor deficits and recovery in each of the different groups. Notice the decreased neuroscore values 2 days after surgery in TBI_E_ and TBI_H_, as well as Sham_E_ rats, but not Sham_H_ rats. TBI_H_ rats neuroscore returned to baseline values faster than the TBI_E_ and Sham_E_ rats. Data are presented as mean ± SD. All the data were compared vs their baseline value (i.e., Day 0 score). ^&&&^*p* ≤ 0.001 vs. Sham_E_ baseline value; ^##^*p* ≤ 0.01, ^###^*p* ≤ 0.001 vs. TBI_H_ baseline value; *p ≤ 0.05, **p ≤ 0.01, ***p ≤ 0.001 vs. TBI_E_ baseline value, Bonferroni test corrected for multiple comparisons.

Assessment of sensorimotor function in craniectomized only rats revealed that there were relatively stable neuroscore values in Sham_H_ rats throughout the duration of monitoring (*p* > 0.05, Fig. 5). As expected, neuroscore values in TBI_H_ rats significantly decreased 2 days postinjury (*p* < 0.001; Fig. 5), started to recover toward baseline values at 7 days, and thereafter were like Sham_H_ rats. By contrast, in Sham_E_ and TBI_E_ rats, neuroscore values were significantly lower than Sham_H_-injured rats at all time points, especially during the first 14 days after TBI (*p* < 0.05; Fig. 5).

### Acute electrographic post-traumatic seizures

**FIG. 6.**
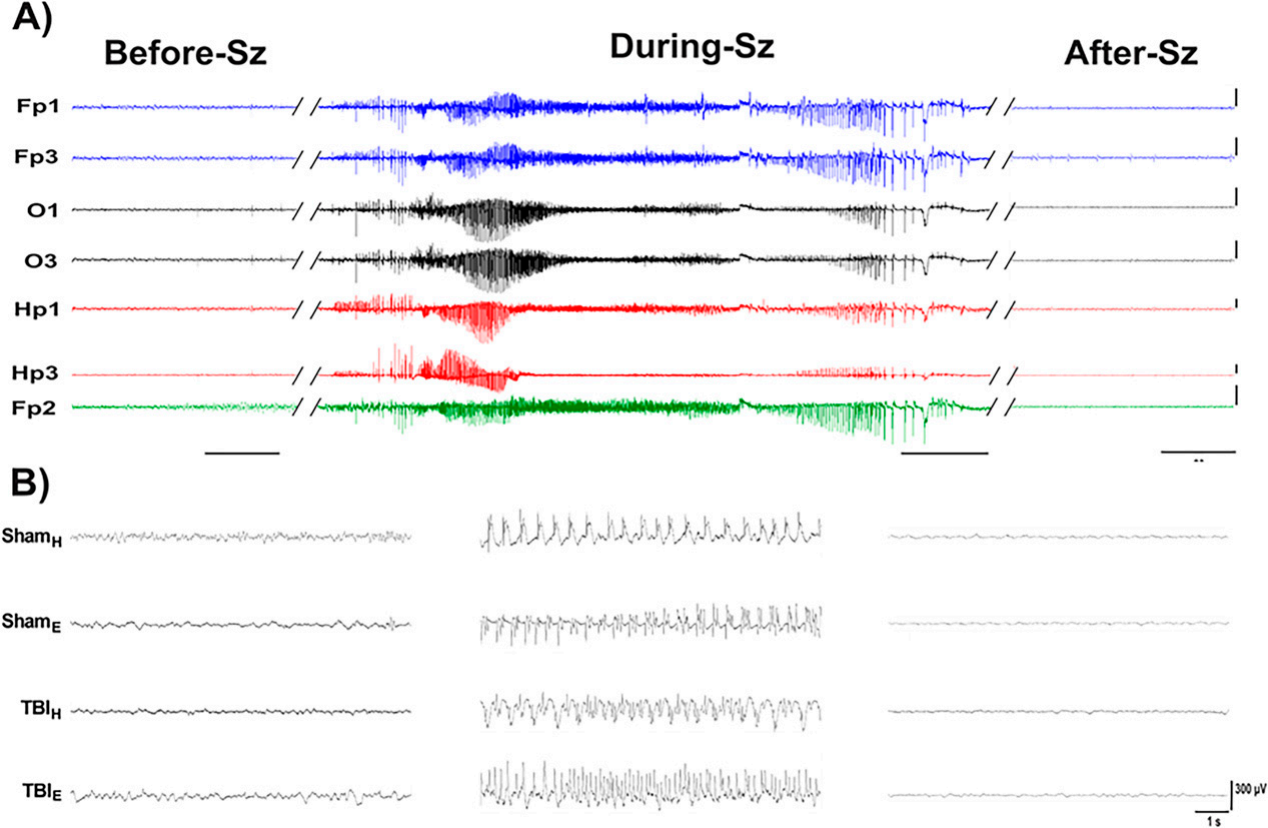
Acute electrograph seizures recorded from rats in each craniectomy group. **(A)**: Example of acute generalized seizure recorded from one TBI_H_ rat 2 days after surgery (EEG; 5-min epoch, high-frequency filter 90 Hz). Scale bar = 500 µV in each trace. **(B)** Expanded 10 s traces from a single channel (Fp1) taken 1 min before the seizure onset (Before-Sz), at the mid-point of seizure (During-Sz) and 1 min after the seizure offset (After-Sz). Sz; Seizure.

**FIG. 7.**
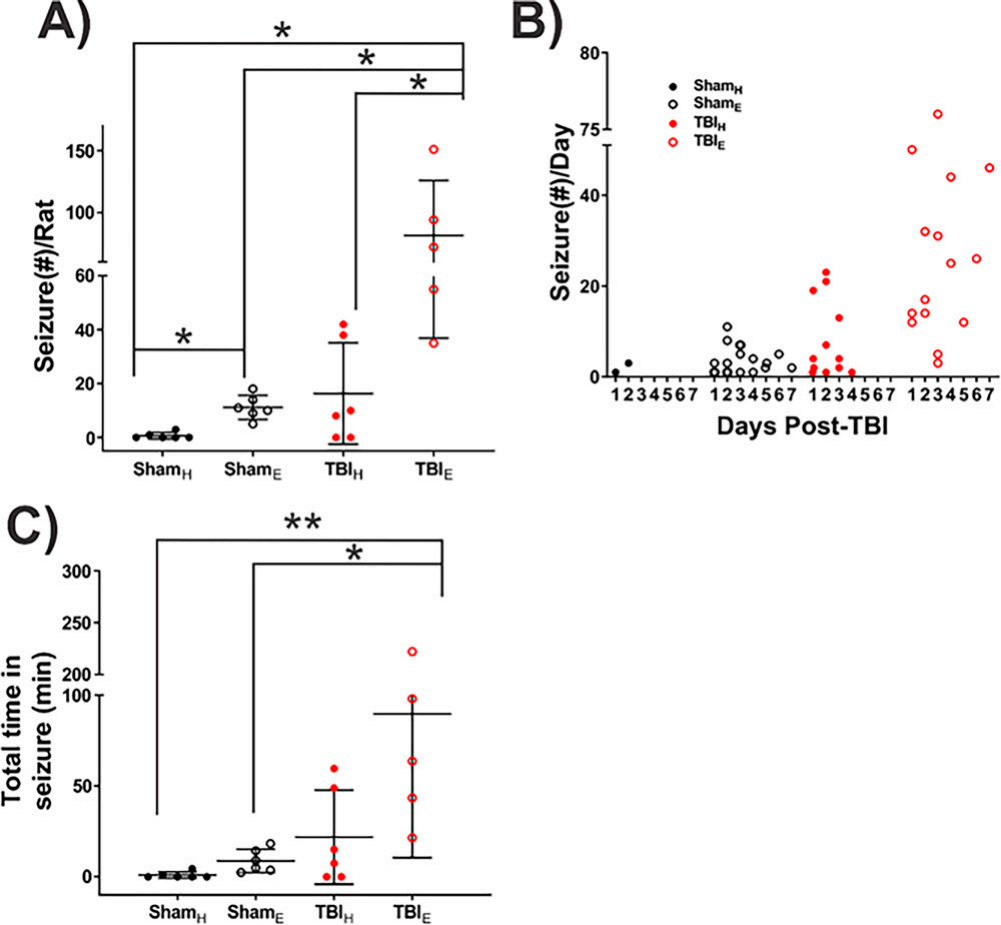
Quantification of acute EEG seizures during the first seven days after TBI **(A)** Plot showing the number of seizures per rat after TBI. TBI_E_ rats had significantly more acute EEG seizures than TBI_H_ and Sham_H_ rats. **(B)** Number of EEG seizures per day for first 7 days. Sham_E_ and TBI_E_ rats had acute EEG seizures all 7 days, but Sham_H_ and TBI_H_ rat seizures resolved 2 and 4 days after surgery respectively **(C)** TBI_E_ rats spent significantly more time having seizures than Sham_E_ and Sham_H_ rats, but similar amount of time in seizures as TBI_H_ rats. Data are presented as mean ± SD. Statistical significance: *p < 0.05; **p < 0.01; one-way ANOVA test followed by *post hoc* analysis with Bonferroni.

EEG during the first 7 days after surgery recorded electrographic seizures, consisting of repetitive epileptiform discharges that evolved in frequency, amplitude, and morphology of discharges, and regularly ended with postictal amplitude suppression (Fig. 6). Two of six Sham_H_ rats had a total of four seizures within 48 h after surgery and none thereafter. In these two rats, the mean latency to the first electrographic seizure was 19.5 ± 16.1 h, the mean seizure duration was 83.3 ± 35.5 s, and the total time spent having electrographic seizures was 2.8 ± 1.6 min (Table 2, Fig. 7).

**Table 2.** Acute Electroencephalograph Seizures After TBI

Group	EEG seizure activity	Latency (h)	Cumulative seizure	Seizure duration (sec)
Sham_H_	2/6 (33.3%)	19.5 ± 16.1	4	83.3 ± 35.5
Sham_E_	6/6 (100%)^*^	27.3 ± 5.6	67	35.8 ± 29.3
TBI_H_	4/6 (66.6%)	12 ± 1.6	98	79.9 ± 56.5^***^,****
TBI_E_	6/6 (100%)^*^	29.6 ± 39.1	407	63.9 ± 42.9^**^

^*^ *p* ≤ 0.5 vs. Sham_H_, chi-square test.

^**^ *p* ≤ 0.01.

^***^ *p* ≤ 0.05 vs. Sham_E_.

^****^p ≤ 0.05 vs. TBI_E_; one-way ANOVA–Bonferroni.

All six Sham_E_ rats (*p* < 0.05, compared with Sham_H_ rats) had seizures and overall 67 seizures were recorded. The average number of seizures per Sham_E_ rat was significantly higher than in Sham_H_ rats (11.1 ± 1.8 vs. 2.0 seizures; *p* < 0.05). Unlike the Sham_H_ rats, Sham_E_ rat seizures were detected during the entire first week of recording. The mean latency to the first seizure was 27.3 ± 5.6 h, the mean seizure duration was 35.8 ± 29.3 s, and the total time having electrographic seizures was 8.7 ± 2.6 min (Table 2, Fig. 7).

Four out of six TBI_H_ rats had a total of 98 seizures during the first 96 h after LFPI and none in the remaining 72 h of the recording. The mean latency to the first seizure was shorter than latencies in Sham_H_ and Sham_E_ rats (12 ± 1.6 h), mean seizure duration was 79.9 ± 56.5 s, and total time having seizures was 32.7 ± 12.7 min (Table 2, Fig. 7).

All six TBI_E_ rats had seizures (*p* < 0.05, compared with Sham_H_ group) that totaled 407 seizures during the first week of recording. There was a greater number of seizures per rat (81.4 ± 19.9 seizures), longer mean seizure duration (63.9 ± 42.9 s; *p* < 0.05), and longer amount of time spent having electrographic seizures in TBI_E_ rats than in Sham_H_ and Sham_E_ rats (89.7 ± 35.4 min; Table 2, Fig. 7).

## Discussion

The current study found greater MRI positive tissue deformation at 9 days and in most cases, weaker functional connectivity in the ipsilateral hemisphere up to 7 days after a craniectomy performed with an electrical drill than a hand drill in sham-injured rats. In TBI rats with an electric-drilled craniectomy, rats had prolonged sensorimotor deficits and greater number of acute seizures than TBI rats with hand-drilled craniectomy. Results suggest an electrical drill craniectomy can produce harmful brain structural and functional changes that worsen behavioral outcome and acute seizures after LFPI. Thus, the craniectomies conducted with an electric drill can constitute an injury itself, which will doubtless reduce the effect size when comparing TBIs with sham controls.

Studies in rabbits found that a craniectomy performed with electrical drill generates local heat that could be transmitted to the surface of the brain.^26^ The heating and vibration from the electric drill could disrupt blood vessels and nerves, initiating an immediate primary neurovascular injury, followed by a more severe secondary biochemical cascade producing local cytotoxic edema.^17,26^ The effects of heating could be reduced by continuous application of cold saline solution, regular breaks between drilling periods, and low drilling speeds combined with safest feed rates that minimize the risks of penetrating the brain tissue.^18^ However, it’s more difficult to dampen vibration. In contrast, the hand drill method generates the craniectomy by slow, steady, grinding pressure to penetrate the calvaria.

The mechanical stress induced by the electrical drill craniectomy likely contributes to the increased MRI positive tissue deformation after 9d. The tissue deformation could be related to edema, which is one of the primary lesions detected after injury and only partially resolves 7 days postinjury.^27–30^ Whether edema is complication of intracerebral hemorrhage (ICH) and consequent increase in intracranial pressure is unclear,^30^ though none of the animals showed signs of ICH (e.g., unresponsiveness to stimuli, immobility, lateral recumbent position, significant weight loss, etc.), but many had seizures. Earlier studies found T2 evaluation of the cortical lesion 3 days after the injury is a sensitive parameter to predict severity of histologically verified cortical neurodegeneration and functional impairment.^24^ Thus, the acute MRI changes in animals with an electric-drilled craniectomy could forecast long-term structural changes such as gliosis, neuronal cell loss, and synaptic reorganization. More recent reports showed the craniectomy procedure in sham animals induced the overexpression of endothelial tyrosine kinase,^16^ increase of KC-GRO and IFN-γ cytokines,^17^ and imbalance in the pyruvate dehydrogenase kinase and pyruvate dehydrogenase phosphatase, which often correspond with alterations in the glucose metabolism and cellular energetics.^31^ These latter changes could contribute to long-term morphological brain damage, impaired sensorimotor responses, and possibly increased brain excitability in sham animals.^17^

Severity of brain injury in animals with TBI also is assessed with sensorimotor tests similar to the neuroscore.^32^ In the current study, we expected TBI rats to have lower neuroscore values that represent sensorimotor impairment 2d postinjury.^13^ However, TBI_H_ rats recovered faster than TBI_E_ rats, and unexpectedly animals with electric-drilled craniectomy—TBI and sham—had the longest recovery and neuroscore values that were below baseline values 28 days after injury. Consistent with the MRI results, differences in neuroscores between animal groups suggest an electric-drilled craniectomy could produce more severe, widespread injury associated with functional deficits.

We performed an EEG analysis using GEC, which previous studies showed was a reliable and stable measure of EEG connectivity areas,^33,34^ and recent work suggests the increased strength of GEC correlates with increased synchrony of inhibitory activity.^35^ In the current study, overall, there was weaker GEC in Sham_E_ rats than Sham_H_ rats. One interpretation of these results electrical drilling produces neuronal injury that includes a decrease in the synchrony of inhibitory activity.^36,37^ If this is correct, then a consequence of decreased inhibitory synchrony could be increased cortical excitability. Results from our analysis of early seizures support this interpretation since we detected the greatest number of seizures in TBI_E_ rats and the number of rats with seizure and the number of seizures was comparable between Sham_E_ and TBI_H_ rats. The lowest number of seizures was in the Sham_H_ rats. In this latter group, we detected seizures in two rats, and this could be due to the hand-drilled craniectomy, although we cannot exclude the possibility that electrode implantation could induce acute injury and increased excitability.^38,39^ There is the implication of choice of craniectomy versus anesthetic control shams, which is especially relevant in studies of TBI. If extra-steps are not taken with electrical drilling, then an additional injury as described in the current study could confound studies of TBI, such as studies relating changes in MRI, electrophysiology, or behavior with severity of injury, which often is equated with force of impact or angle of pendulum. In our study, neuroscore appeared a good measure of total injury severity when using electric-drilled craniectomy. Also, choosing to control for the effect of craniectomy after LFPI by using shams with a craniectomy could affect biomarker studies of post-traumatic epilepsy (PTE). For example, an increase in acute seizures due to an injurious craniectomy could increase brain pathology and activity-dependent molecular changes that are related to craniectomy and not the TBI.

## Abbreviations Used

ANTs: Advanced normalization tools
AP: Anterior-Posterior
BL: Baseline
DV: Dorsal-Ventral
EDF: European data format
EEG: Electroencephalograph
FDR: False discovery rate
Fp: Frontoparietal
GEC: Gamma event connectivity
GND: Ground
H: Hippocampus
ICH: Intracerebral hemorrhage
IFN-γ: Interferon gamma
KC-GRO: Keratinocyte chemoattractant-human growth-regulated oncogene
LFPI: Lateral fluid percussion injury
ML: Medial-Lateral
MRI: Magnetic resonance imaging
O: Occipital
PTE: Post-traumatic epilepsy
REF: Reference
ShamE: Sham electric-drilled
ShamEMRI: Sham electric-drilled MRI
ShamH: Sham hand-drilled
ShamHMRI: Sham hand-drilled MRI
Sz: Seizure
TBI: Traumatic brain injury
TBIE: TBI electric-drilled
TBIH: TBI hand-drilled
